# Supplementary material for: Three-dimensional nonreciprocal transport in photonic topological heterostructure of arbitrary shape
Source: Sci Adv. 2025 Jan 8;11(2):eadq9285. doi: 10.1126/sciadv.adq9285 (PMC13108744; doi:10.1126/sciadv.adq9285)
Supplement: Supplementary file 1 — Supplementary Text Figs. S1 to S11 [file sciadv.adq9285_sm.pdf]

Science Advances
AAAS

Supplementary Materials for

Three-dimensional nonreciprocal transport in photonic topological heterostructure of arbitrary shape

Mudi Wang *et al.*

Corresponding author: Mudi Wang, mudiwang@ust.hk; Che Ting Chan, phchan@ust.hk

Sci. Adv. **11**, eadq9285 (2025)
DOI: 10.1126/sciadv.adq9285

This PDF file includes:

Supplementary Text
Figs. S1 to S11

Supplementary Text

Contents

1 The experimental measurements.

2 Analyzing the cross-shaped heterostructure using a model Hamiltonian.

3 Comparison between 3D non-reciprocal waveguide states and interface states.

1 The experimental measurement.

Fig. S1A and B depict the copper-plated boards placed at the bottom and top, respectively, to prevent electromagnetic wave leakage into the air. Additionally, the upper board is perforated with multiple circular holes, enabling the measurement of the internal field distribution of the sample.

The bottom panel of Figure 2A shows the insertion of 20 aluminum rods (with a diameter of 3mm and a height of 6mm) into the air holes of domain B in the linear heterostructure ABC.

The bottom panel of Figure 3A shows an image of a cross-shaped heterostructure that exhibits periodicity along the *x*-direction. The width of domain B in *y*-direction varies along the *z*-direction. In the first to fifth and eleventh to fifteenth layers along the *z*-direction, the width of domain B is $\frac{\sqrt{3}a}{2}$. In the sixth to tenth layers, the width of the domain B is $\frac{7\sqrt{3}a}{2}$.

The bottom panel of Figure 3D depicts a line-focused sample that exhibits periodicity along the *z*-direction. In the first 15 periods along the *x*-direction, the width of domain A, B, and C are all $\frac{7\sqrt{3}a}{2}$ in the *y*-direction. In periods 16-26 along the *x*-direction, the width of domain A is $\frac{11\sqrt{3}a}{2}$ in the *y*-direction, while the width of domain C is $5\sqrt{3}a$ in the *y*-direction.

The bottom panel of Figure 3G shows the sample of 90-degree curve configuration while undergoing deformation, which has no periodicity in the three directions of *xyz*.

In experiments, we place two probes (Probe 1 and Probe 2) in the sample, where S21 (S12) is the signal received by Probe 2 (1) divided by the signal transmitted by Probe 1 (2). We assume the unit lengths along the x, y, and z directions are a, $\frac{\sqrt{3}a}{3}$ and h respectively.

In the straight sample (Fig. 2A), Probe 1 is positioned at (1, 15.5, 8), while Probe 2 is positioned at (22, 15.5, 8); In Fig. S6, we have altered the position

of the Probe 2, yet all the results continue to demonstrate non-reciprocity. This indicates that non-reciprocity is independent of the specific probe locations, as long as both probes are within domain B.

We kept probe 1 positioned at (1, 15.5, 8). For the crossed-shape sample (Fig. 3A), probe 2 was placed at (21, 12.5, 6). For the focused sample (Fig. 3D), Probe 2 is positioned at (22, 13.5, 9). For the bent and deformed sample (Fig. 3G), Probe 2 is positioned at (20, 6.5, 7).

To explore the impact of non-uniform obstacles in the z-direction on experimental outcomes, we replaced the uniformly tall aluminum rod depicted in the lower part of Fig. 2A with aluminum rods of various heights. These included 4 rods at 2mm, 4 rods at 3mm, 4 rods at 5mm, and 4 rods at 6mm heights. Subsequently, we measured their transmission, with the results illustrated in Fig. S7, which indicates that within the frequency range of TNWS, the sample still exhibits significant non-reciprocal.

2 Analyzing the cross-shaped heterostructure using a model Hamiltonian

Let us consider a heterostructure ABC, where the *x*-direction exhibits periodicity, and the shape in the *yz*-direction is arbitrary (with the condition that domain B is positioned between domains A and C). The structure extends in the *z*-direction with a height of $h_0 = N\mathrm{a}$, and rigid boundaries are present at $z = 0$ and $z = h_0$. For this configuration, the Hamiltonian around K-H valley at $k_x = 0$ can be expressed as $H_{yz} = \begin{pmatrix} m(y,z) & v_D \partial y \\ -v_D \partial y & -m(y,z) \end{pmatrix}$, where $m(y,z)$ is a function of y and z. Here, we have assumed that d_z^j does not exhibit dispersion along the k_z direction in each crystal (as approximately observed in Figure 2E). Moreover, it is evident that as y approaches $\pm\infty$, $m(y,z)$ becomes constant. At $\delta\omega = 0$, by solving the eigen equation

$$H_{yz}\begin{pmatrix} \psi_1 \\ \psi_2 \end{pmatrix} = 0, \qquad (3)$$

we can get the general solution of the second component:

$\psi_2 = C_1(z)e^{\frac{1}{v_D}\int_0^y dy' m(y',z)} + C_2(z)e^{-\frac{1}{v_D}\int_0^y dy' m(y',z)}$. The solution of this equation as y approaches $\pm\infty$ is given by $\lim_{y\to\pm\infty}\psi_2 = C_1(z)e^{\frac{1}{v_D}m_\pm y} + C_2(z)e^{-\frac{1}{v_D}m_\pm y}$. Since $m_+ m_- = d_z^A d_z^C < 0$ ($m_+ = d_z^A < 0, m_- = d_z^C > 0$), there exist confined solutions. The eigen solution cannot diverge at the infinity, hence $C_2(z)$ equals 0 and

$$\psi_2 = C_1(z)e^{\frac{1}{v_D}\int_0^y dy' m(y',z)}. \tag{4}$$

The finiteness of the crystal in the *z* direction, requires that the fields outside the *N* stacking layers reduces to zero, which requires

$$C_1(z) = \begin{cases} 0,\ z \le -\frac{a}{2} \\ f(z), -\frac{a}{2} < z < h_0 + \frac{a}{2} \\ 0,\ z \ge h_0 + \frac{a}{2} \end{cases}, \tag{5}$$

where $z = -a/2$ and $z = h_0 + a/2$ represent the center coordinates of two imaginary outmost layers located at the lower and upper sides of the crystal. Expanding $C_1(z)$ into a Fourier series, we can obtain: $C_1(z) = \sum_{n=0}^{\infty} a_n e^{i\frac{2\pi n}{h_0+a}z}$, where $k_z = \frac{2\pi n}{h_0+a} = \frac{2\pi n}{(N+1)a}$ and n is the number of solutions. Since k_z is a reciprocal lattice vector, it must satisfy:

$$0 \le k_z < \frac{2\pi}{a}. \tag{6}$$

Then we can get $0 \le n < N+1$, and hence there are $N+1$ Fourier components. In addition, we see that the boundary conditions at the two boundaries $z = -a/2$ and $z = h_0 + a/2$ lead to an identical constraint condition $C_1\left(-\frac{a}{2}\right) = C_1\left(h_0 + \frac{a}{2}\right) = \sum_{n=0}^{N+1} a_n = 0$, resulting in a total of *N* independent solutions. According to equation (3-4), we can get $\psi_1 = -\frac{v_D}{m(y,z)}\partial_y \psi_2 = -\psi_2$ and $\psi = \begin{pmatrix}\psi_1 \\ \psi_2\end{pmatrix} = \begin{pmatrix}1 \\ -1\end{pmatrix} C_1(z)e^{\frac{1}{v_D}\int_0^y dy' m(y',z)}$. When $k_x \ne 0$, the perturbation of the Hamiltonian reads $\delta H = k_x \sigma_x v_D$. According to the standard perturbation theory, we acquire the eigenfrequency of the waveguide modes (slightly away from $\delta\omega = 0, k_x = 0$)

$$\delta\omega = \frac{<\psi|\delta H|\psi>}{<\psi|\psi>} = k_x v_D, \tag{7}$$

which means the N independent solutions have the same dispersion. Based on these, there are 15 TNWSs at each valley for the structure shown in Fig. 3a, and their slopes satisfy equation (7), since it consists of a stack of 15 layers in the z-direction.

3 Comparison between 3D non-reciprocal waveguide states and interface states.

Fig. S8A,B depict ABC and AC heterostructures (with periodicity in the x-direction), respectively, with multiple 90-degree bends. In domain A, the magnetic field is oriented along the -z direction, while in domain C, it is oriented along the +z direction. Domain B, on the other hand, does not have a magnetic field. Figs. S8C,D show the projected band structures corresponding to these two heterostructures, respectively. The field distributions of the eigenstates of 15 TNWSs at $k_x = -0.65\pi/a$ in Fig. S8C (Fig. S8D) are shown in Fig. S9 (Fig. S10). When the eigenmodes of the 15 waveguide states at $k_x = -0.65\pi/a$ for the ABC structure are added together, the resulting field distribution spans the entire domain B and forms a connected pattern, as shown in Fig. S11A. While the eigenmodes of the 15 waveguide states at $k_x = -0.65\pi/a$ for the AC structure are summed, the resulting field distribution occurs at the interface of AC along the Chern vector direction (001) and appears to be some separated patterns, as shown in Fig. S11B. It is noteworthy that for the ABC (AC) configuration, the number of waveguide modes (boundary modes) depends only on the number of layers along the (001) direction, aligned with the Chern vector direction. Interfaces also exist along the (010) direction, but they are perpendicular to the direction of the Chern vector and thus do not play a role in supporting edge modes.

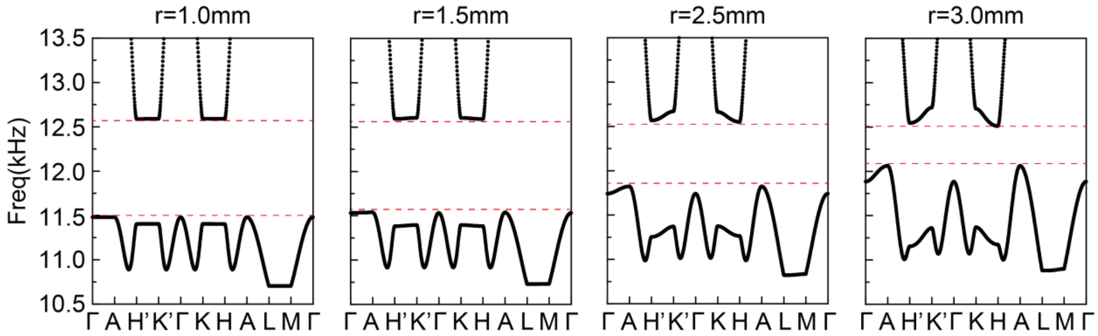

Fig. S1. **The impact of coupling strength on band structures.** Band diagrams of the photonic crystal for Crystal A with r=1.0, 1.5, 2.5 and 3.0 mm.

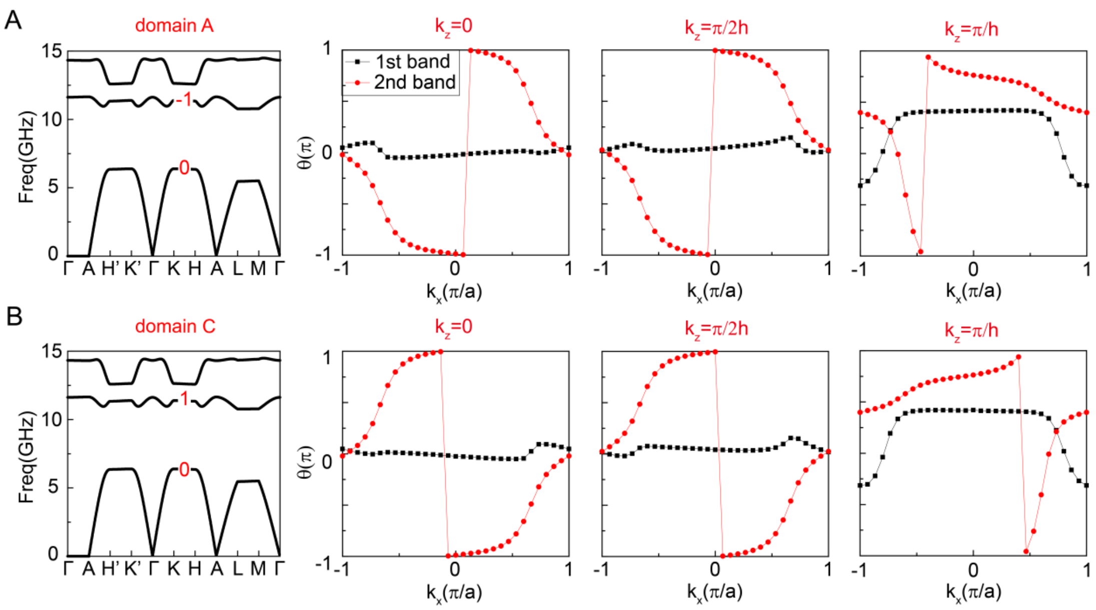

Fig. S2. **Calculation of the Chern number through full-wave simulation.** A,B, The band structure for domain A (C), and the corresponding evolution of the Berry phase of two bands under the second gap (around 12GHz) at $k_z = 0$, $k_z = \frac{\pi}{2h}$ and $k_z = \frac{\pi}{h}$.

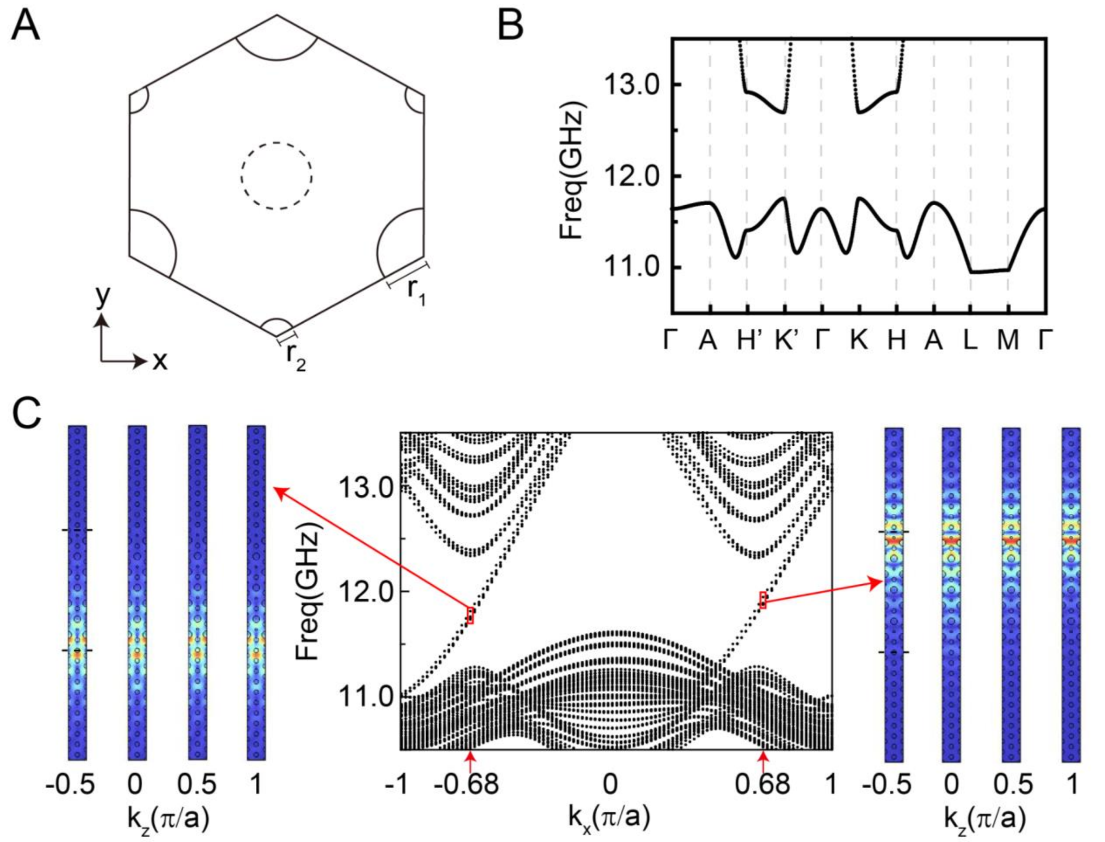

Fig. S3. **ABC heterostructure when domain B is a crystal with a trivial gap.** **A**, Top view of the unit cell with with $r_1 = 3.0\text{mm}$ and $r_2 = 1.0\text{mm}$, with no magnetic field. **B**, The band structure of the unit cell shown in **A**. **C**, Band diagram of the ABC heterostructuren with $k_z = -\frac{\pi}{2h}$, 0, $\frac{\pi}{2h}$, $\frac{\pi}{h}$, where crystal B represents the nodal line in **A**, along with the field distribution of the eigenstates.

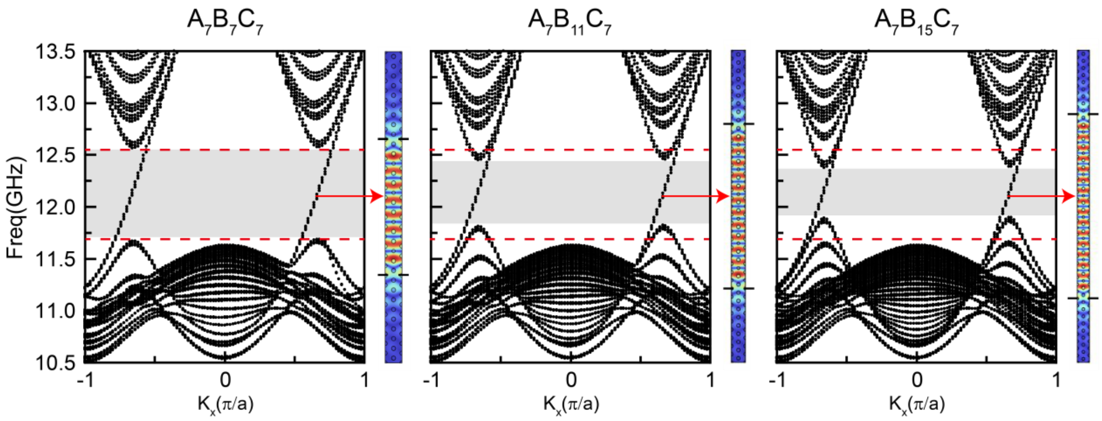

Fig. S4. **Projected bands of the heterostructure when the width of domain B takes three different values.** The projected bands along k_x for $A_7B_7C_7$, $A_7B_{11}C_7$ and $A_7B_{15}C_7$ structure with $k_z = -\frac{\pi}{2h}$, 0, $\frac{\pi}{2h}$, $\frac{\pi}{h}$ (the projected bands with different k_z are plotted on the same graph). In each figure, the gray area represents the frequency range of TNWSs, while the two black dashed lines indicate the gap regions of Crystals A and C.

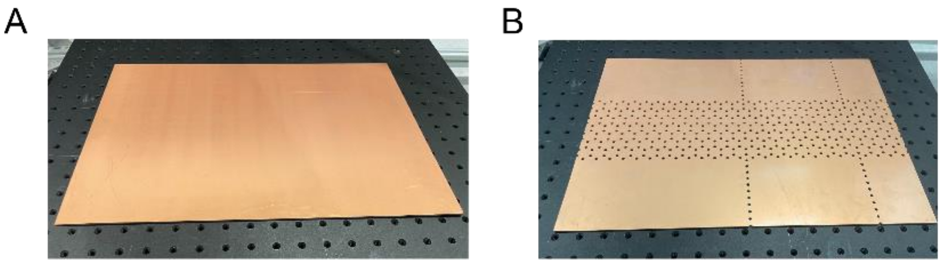

FIG. S5. **Photographs of the PEC boards. A,B,** The photograph of the copper-plated boards positioned at the bottom and top.

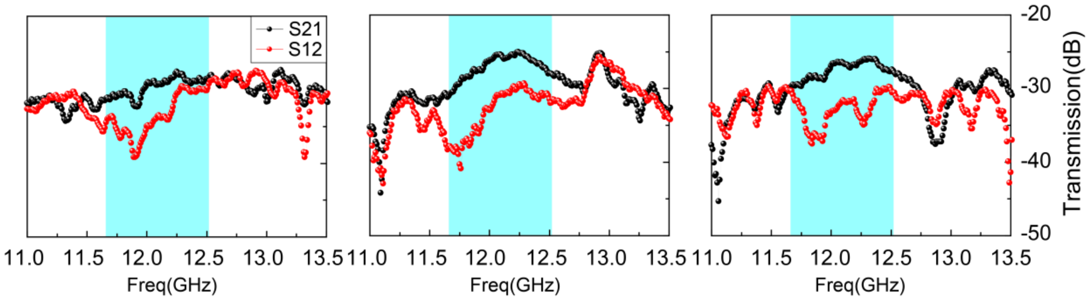

Fig. S6. **The impact of measurement position on the transmission.** Transmission coefficients S12 and S21 for the straight line configuration, with Probe 2 positioned at three different locations: (22, 13.5, 7), (20, 17.5, 5) and (22.5, 12, 10).

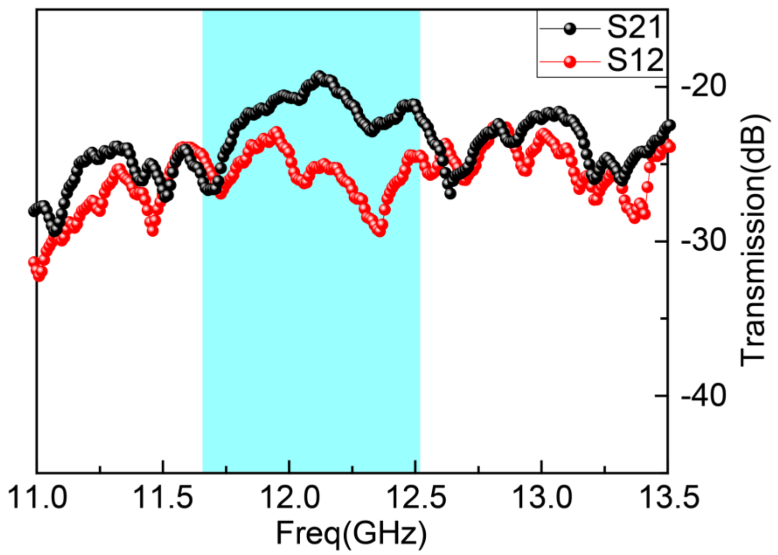

Fig. S7. **Transmission of heterostructure with non-uniform defects in the z-direction.** Transmission coefficients S12 and S21 for the structures with 20 aluminum cylinders with different length (including 4 each of aluminum rods with heights of 2mm, 3mm, 4mm, 5mm, and 6mm, while the diameter is 3mm).

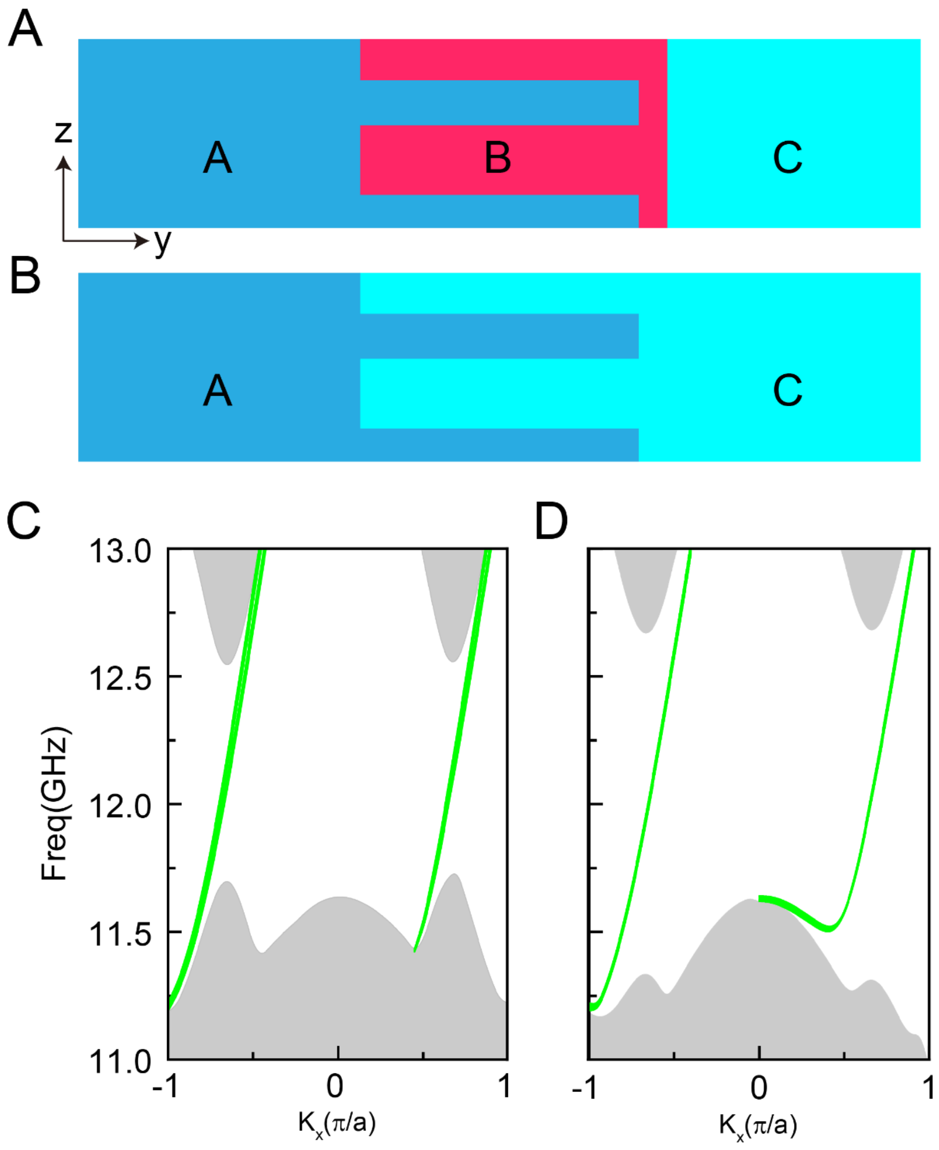

Fig. S8. **Comparison of projected band structures between the ABC and the AC heterostructure. A,B,** The shape of the ABC (**A**) and AC (**B**) heterostructure with multiple 90-degree bends. **C,D,** The projected band of ABC (**C**) and AC (**D**) structure.

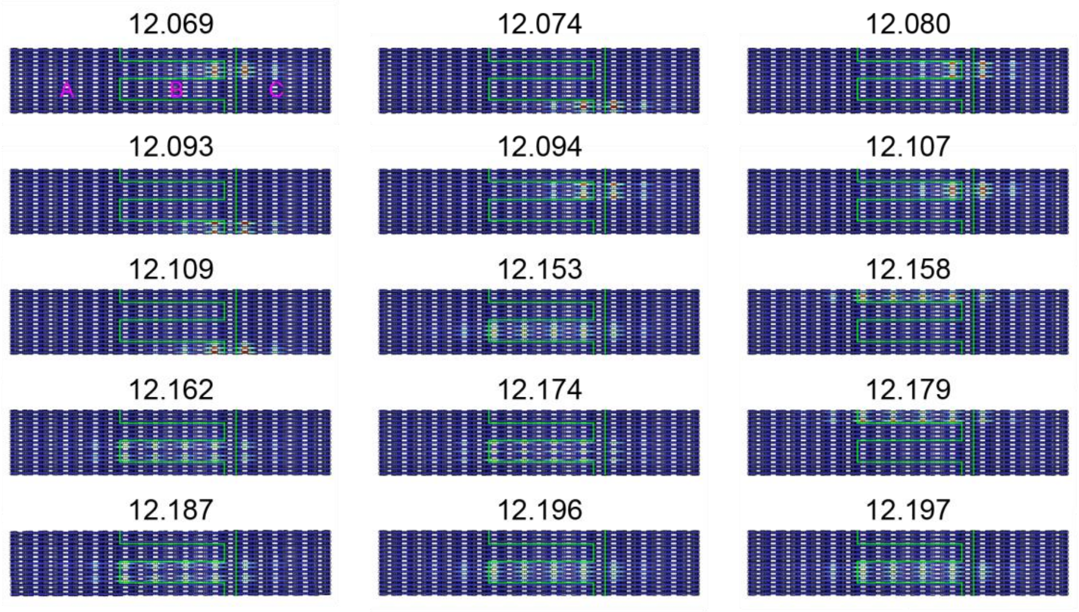

Fig. S9. **Field distribution of the ABC heterostructure.** The field distributions of the 15 eigenstates of ABC heterostructure at $k_x = -0.65\pi/a$.

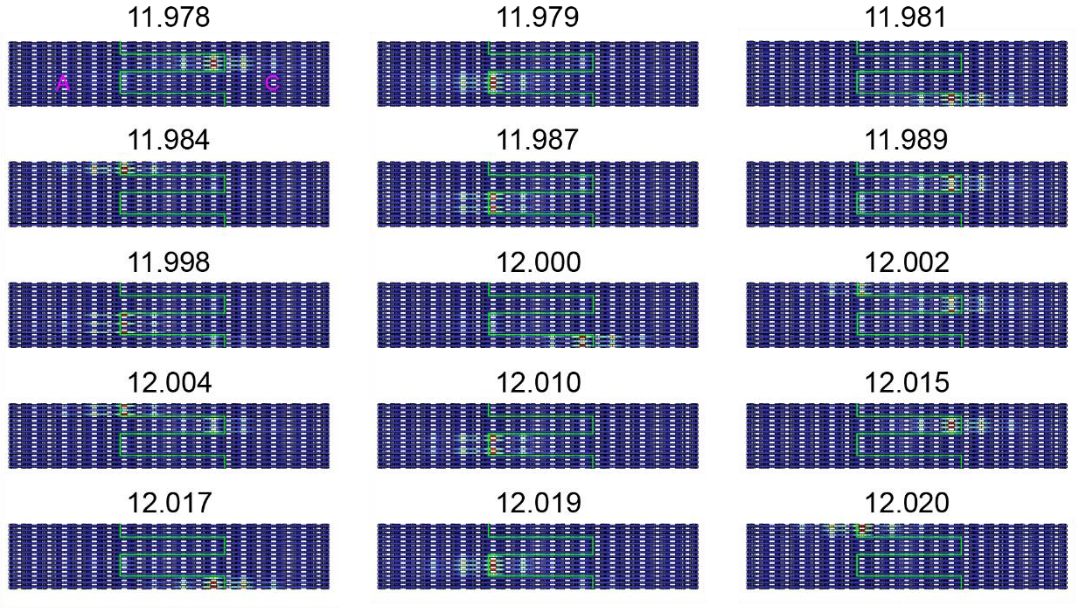

Fig. S10. **Field distribution of the AC heterostructure.** The field distributions of the 15 eigenstates of AC heterostructure at $k_x = -0.65\pi/a$.

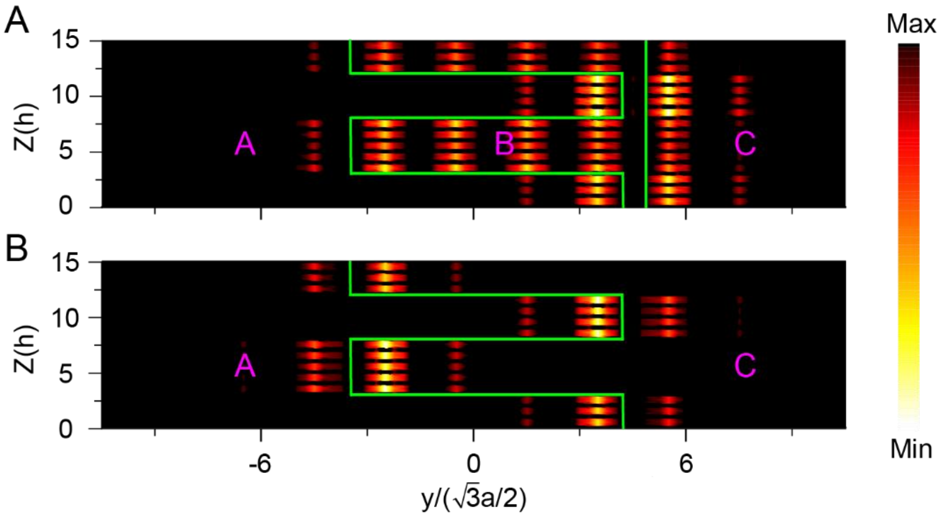

Fig. S11. **Comparison of field distributions between the ABC and AC heterostructure.** The sum of the field distributions of the 15 eigenstates of ABC (**A**) and AC (**B**) heterostructure at $k_x = -0.65\pi/a$.
